# A study of the impact of internet use on the mental health of rural older adults—empirical analysis based on China General Social Survey 2021 data

**DOI:** 10.3389/fpubh.2024.1470965

**Published:** 2024-12-13

**Authors:** Jiangwei Hu, Guocai Zhang, Mingda Wang

**Affiliations:** ^1^School of Chinese Literature and Media, Hubei University of Arts and Science, Xiangyang, China; ^2^School of Education, National University of Malaysia, Bangi, Selangor, Malaysia

**Keywords:** internet use, rural older adults, mental health, CGSS, social participation

## Abstract

**Background:**

In the context of the era of both digitalization and aging, Internet use plays an important role in supporting the rural older adults to actively integrate into the digital society and improve their mental health.

**Purpose:**

To explore the impact of Internet use on the mental health of rural older adults and the mediating role of their social participation.

**Methods:**

Based on the latest data from the China General Social Survey (CGSS) 2021, the study utilized linear regression analysis to explore the impact of Internet use on the mental health of rural older adults and the mediating role of their social participation.

**Results:**

The two important results of the analysis are: (1) internet use is significantly and positively related to the mental health of rural older adults, and (2) internet use can have an indirect effect on the mental health of rural older adults through two pathways: (a) the complete mediating effect of social participation;(b) partial mediating role of friend-gathering type participation.

**Discussion:**

Internet use, social participation, and friend-gathering type participation all have an effect on the mental health of rural older adults. The research results reveal the impact of Internet use on the mental health of rural older adults and its mechanism, which is helpful to provide useful enlightenment for improving the mental health of rural older adults in the Internet era.

## 1 Introduction

In today’s world, where the wave of informatization and digitization is sweeping across the globe, the rapid development and wide application of internet technology is becoming an important engine for social progress. As an important part of economic and social development, the modernization process of rural areas is directly related to the quality and level of the country’s overall development. With the continuous improvement of network infrastructure, the network coverage and quality of network services in rural areas have been significantly improved, and internet access is no longer a remote area for rural older adults, but is gradually integrated into an important part of their daily lives. With the rapid development of the internet industry in recent years (1), internet penetration among rural populations has exploded (2), and is gradually penetrating remote areas and older populations.

Alongside the digital wave is the deepening of global population aging (3), which has become an important feature of the global society due to significant changes in population size, declining fertility rates, and increasing life expectancy. Rural older adults people are an important force for the future development of rural areas, and the active integration of rural older adults people as “digital refugees” into the network society without being reduced to a “digital relic” is related to the re-socialization under the ecology of “active aging.” The positive integration of rural older adults groups into the network society as “digital refugees” without being reduced to “digital leftovers” is related to the resocialization behavior under the ecology of “active aging” and the overall mental health of the older adults.

Health is a multidimensional concept that covers physical, mental and social adaptation, and mental health is often overlooked compared to physical health. Mental health, on the other hand, is not only related to the individual’s sense of well-being and quality of life, but also closely related to the stability and development of society. The mental health of the older adults in today’s society is not optimistic (4), and a large number of rural young and middle-aged laborers have left the countryside, so the phenomenon of rural “empty nesters” is serious, and has become the object of social attention. As a special group of the older adults, “empty nesters” are aging, declining in organizational function, and may experience life changes such as widowhood and living alone, while facing challenges such as intergenerational separation and digital divide (5, 6). All these difficulties contribute to many psychological problems such as depression, loneliness, and dementia among rural older adults (7, 8), and the older adults also develop more negative emotions due to increased loneliness and depression (9, 10).

The concept of “active aging” is a scientific and effective ideology that has been developed to cope with the aging of the population worldwide. The process of “active aging” is a process of participation by the whole society. “Participation,” as one of the three pillars of active aging, refers to “the participation of older people in socioeconomic, cultural and spiritual activities according to their abilities, needs and preferences” (11). With the advent of the digital economy, the internet is reshaping individuals’ life patterns and social relationships in unprecedented ways (12, 13). The booming development of the internet industry has also provided rich and diversified entertainment for the older adults in their later years, and has become an important standard of practice for the social participation of the rural older adults, which subsequently expands the intensity of social participation of the older adults (14). The use of the internet can serve as a resource tool for rural older adults people to socialize and entertain themselves and learn knowledge, and it can also help older adults people to reduce psychological barriers such as depression and loneliness (15–17), which is also a profound realization of the concept of “active aging.” Older adults who use the internet more frequently and appropriately have greater participation in social activities and better mental health (18–20). Internet use influences the lifestyles and attitudes of rural older adults, and its popularization in rural areas can help rural older adults obtain information, amusement and entertainment, enrich their daily activities, and to a certain extent improve their mental health and ultimately improve the quality of life of rural older adults (21, 22).

It is worth noting that most of the current academic research on the effect of internet use on the mental health of the older adults focuses on the exploration of the internet use of the older adults and their health, life satisfaction, subjective well-being and other factors, with less attention paid to the micromechanisms of the internet use of the mental health of the older adults, and the selection of the research object focuses on the older adults as a whole, with less discussion of the rural older adults and the analysis is only as a factor in the heterogeneity test, and few studies have focused on exploring the mental health mechanisms of internet use on the rural older adults as a specific group. Therefore, in view of the existing research in the academic community, the article takes the research of multiple scholars on the relationship between older adults internet use and their mental health as a macro perspective, and takes rural older adults as a micro individual as the central perspective, to analyze the effects and internal mechanisms of internet use on the mental health of rural older adults. The Chinese General Social Survey (CGSS) is a representative of continuous cross-sectional social surveys in China, which provides a comprehensive and systematic description and analysis of Chinese society through annual survey data, revealing the development trends of various aspects and levels of social change in China. The survey data is publicly released through the official website of the China Research and Data Center of Renmin University of China^1^. Users can download and use the latest CGSS 2021 survey data for free through registration and application http://cgss.ruc.edu.cn/. Based on the CGSS2021 data, we screened a separate sample of rural older adults people, used linear regression analysis to specifically explore the effect of rural older adults people’s internet use on their mental health, and conducted an indepth empirical analysis of the mechanism by which social participation mediates the relationship between rural older adults people’s internet use and their mental health.

## 2 Literature review and research hypotheses

### 2.1 Older adults’ internet use and mental health

Currently the internet is widely used globally and has become a daily behavioral tool for communication for many people including older adults (23). Hong et al. (24) used structured interview method to investigate the impact of internet use on the mental health of older adults people, and found that older adults people who use the internet can feel higher social connections and have positive emotional psychology toward society. Sum et al. (25) investigated the relationship between the use of the internet and the sense of community among the older adults in Australia through exploratory research methods, and found that the internet can significantly enhance the sense of community among the older adults in Australia, namely, the sense of community, happiness and life satisfaction, and improve the mental health level of the older adults. Chen and Persson (26) used questionnaires to survey older adults users in northwestern Ohio, the United States, and found that older internet users were more active and healthy in terms of mental health and personal characteristics than non-internet users. Shapira et al. (27) used a quasi-experimental research design to find that internet use helps older adults people improve their sense of happiness and empowerment, promote their cognitive function and independent experience. Kim et al. (28) used the quota sampling method to investigate the impact of mental health services for the older adults in South Korea, and found that internet websites providing mental health knowledge played a positive role in improving the mental health literacy of the older adults. Xavier et al. (29) pointed out in a longitudinal study based on UK aging data that British older adults people who frequently use the internet show a higher propensity to prevent cancer. Long et al. (30) used the data analysis of the China Family Panel Study (CFPS 2018) to show that the popularity and application of the internet have shown significant positive effects in promoting the mental health of rural and urban older adults groups. In light of the aforementioned scholarly studies, this paper posits the following hypotheses:

*H1*: Internet use is significantly and positively related to psychological health of rural older adults.

### 2.2 Internet use and social participation of older adults

The internet has provided a new and different avenue for social interaction, allowing groups and relationships to form that would otherwise not come together, and the ability to use the internet has become critical to participation in many areas of society (31). Sillence et al. (32) pointed out from the perspective of information acquisition that the older adults use the internet to actively search for health knowledge and information to make up for their own health knowledge defects, which can strengthen their participation in online service activities, improve their ability to adapt to the network society, and thus promote the social participation of the older adults. Zhang and Li (33) pointed out from the perspective of social network that the use of the internet can improve the social network of the older adults and improve their social participation level. Byun et al. (34) pointed out from the perspective of social relations that the higher the internet use probability of the older adults, the larger their circle of friends and significantly improved their social participation. Heo et al. (35) from the perspective of interpersonal communication of the older adults, pointed out that the use of the internet has increased the frequency of communication between the older adults and others, reconnected the older adults with the outside world, and strengthened communication and cooperation with the outside world. The internet, as a new type of social participation, is a platform for people’s online interaction and communication, and a supportive prerequisite for older people’s offline participation in social activities, which transforms the community and integrates it into the rhythm of daily life, combining online life and offline activities (36), and helps older people to gradually form online and offline combined new patterns of social interaction, expanding older people’s social networks and improving their social integration. In light of the aforementioned academic research, this paper proposes the following hypothesis:

*H2*: Internet use is significantly and positively related to social participation of rural older adults.

### 2.3 Social participation and mental health of older adults

Health is an important goal of active aging, and the mental health of older adults is a key concern of society, while the intensity of older adults’ participation in social activities is also closely related to their state of mind in the re-socialization process. Hong et al. (37) investigated the structural relationship between social participation activities and the longitudinal trajectory of depression among older adults people in Singapore, and found that there was a significant negative correlation between older adults people’s participation in cultural volunteer services, exercise, and sports events and their depression, That is, the greater the intensity of older adults people’s participation in social activities, the lower the level of depression. Croezen et al. (38) found that participation in religious activities can help reduce depressive symptoms in older adults people in Europe. Everard et al. (39) explored the impact of participation in leisure social activities on the functional health of older adults, and found that maintaining low demand leisure activities (such as sewing, reading, watching TV, and listening to music) in older adults leads to a more positive and healthy psychological state, and a corresponding increase in happiness. Kekäläinen et al. (40) conducted a longitudinal study on the association between leisure time participation in sports activities and mental and subjective health, and found that sports activities are an external manifestation of promoting good mental health, and more participation in leisure sports activities indicates mental health. Oh et al. (41) investigated the relationship between exercise type and quality of life in the Korean population with metabolic syndrome (Mets), and found that older adults who participate in exercise more frequently tend to be psychologically healthier. As a vulnerable group in society, the older adults in rural areas are the target of society’s best efforts. Older people who actively participate in various social activities are able to give full play to their re-socialization skills, and the healthier their psychological state will be. Therefore, in view of the above scholars’ research, this paper proposes the following hypotheses:

*H3*: There is a significant positive correlation between social participation of rural older adults and their mental health.

### 2.4 The mediating role of social participation

**Figure 1:**
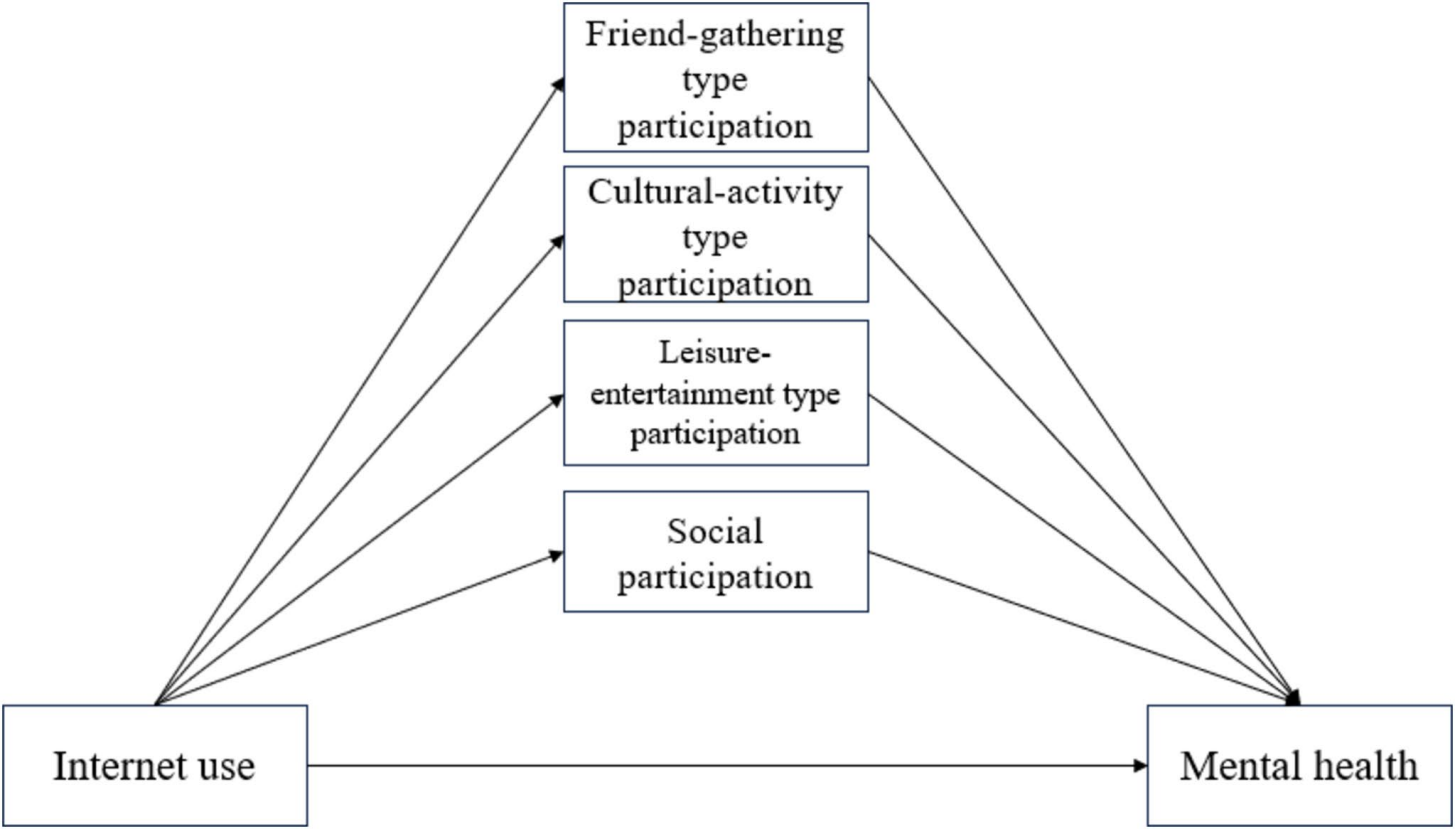
Research hypothesis model.

Internet access plays an important role in supporting the social participation of rural older persons, while at the same time social participation, as a necessary requirement for the continuous promotion of the comprehensive development of older persons, has become an expression of action to maintain the mental health of older persons. Liu et al. (42) explored the potential relationship between internet use and depressive symptoms of middle-aged and older adults people and concluded that internet use would reduce their depression by improving their social participation, Yu et al. (43) analyzed the relationship between the use of online social networking sites (SNS) and the social well-being of older adults people in the United States, and found that using online networks helps older adults people strengthen frequent connections with acquaintances in their daily lives, promote their participation in social activities to obtain different social benefits, and thus improve their life satisfaction. Using the internet to keep in touch with relatives and friends or make new friends can increase the perception of friends’ support and form new social interpersonal relationships (44), help them obtain psychological comfort, help them improve their mental health, and thus maintain a life state of full sense of value. This, in turn, can facilitate the acquisition of psychological comfort and contribute to the enhancement of mental health, thus maintaining a life state characterized by a sense of value. As a result, synthesizing the above academic research, this paper proposes the following hypothesis and the hypothesis model is shown in Figure 1.

*H4*: Social participation mediates the relationship between rural older adults people’s internet use and their mental health.

*H4a*: Leisure-entertainment type participation mediates the relationship between rural older adults internet use and their mental health.

*H4b*: Cultural-activity type participation mediates the relationship between rural older adults internet use and their mental health.

*H4c*: Friend-gathering type participation mediates the relationship between rural older adults internet use and their mental health.

## 3 Materials and methods

### 3.1 Data sources and sample composition

The data for this study comes from the China General Social Survey (CGSS) 2021 survey data. The data have been collected from a continuous cross-sectional survey of more than 10,000 households in all provinces, autonomous regions, and municipalities directly under the central government in mainland China since 2003, and the questionnaire data are rich and authoritative, with a total of 8,148 samples obtained in 2021, which is a good representation of the population. The program provides detailed data support for the study of internet use on the mental health of rural older adults. As the object of the study is the rural older adults, in accordance with the general situation of the study, first of all, the household registration for the agricultural household is defined as rural residents, on this basis, the age of 60 years old and above, a total of 2090 samples, combined with the title of the questionnaire will be part of the question of incomplete answers as invalid value to be given to the exclusion of, and ultimately obtained 2028 valid samples for statistical analysis. The study used the statistical analysis software IBM SPSS Statistics 27, and utilized the survey data from the China General Social Survey (CGSS) 2021, and applied the linear regression analysis method to explore the effect between the rural older adults’s internet use, social participation and their mental health. Results were considered statistically significant when rejecting alpha at *p* < 0.05.

### 3.2 Variable setting

In this study, all variables are divided into explained variables, explanatory variables, mediating variables, and control variables, which are explained and measured as follows.

#### 3.2.1 Explained variables

Currently, the assessment system for the mental health of the older adults mainly uses multidimensional indicators and unidimensional indicators to measure. Self-assessment of health is the most commonly used indicator to reflect an individual’s subjective health status, and some scholars have also commonly used unidimensional indicators (individual self-reporting) as a method to measure the mental health of older adults (45). Therefore, this study draws on the research approach of Xu and Zhang to explain the variable of mental health (46), using a single dimensional health indicator strategy to evaluate the mental health status of the older adults population. Specifically, by asking the question “How often have you felt depressed or down in the past 4 weeks?” We set five gradients of “always, often, sometimes, seldom, and never” for the respondents to choose from, and assigned these options a quantitative score of “1–5” on a five-point Likert scale to reflect the positive changes in mental health, i.e., the higher the score, the higher the mental health status of the rural older adults. The higher the score, the better the mental health of the rural older adults.

#### 3.2.2 Explanatory variables

The explanatory variable of this study is internet use. Drawing on the research idea of Yang et al. (47), the question “In the past year, you have used the internet (including cell phones)” was selected as the measurement of internet use in the CGSS2021 questionnaire, and a five-point Likert scale was used to code the answers, assigning a value of 1 to “Never,” 2 to “Rarely,” 3 to “Sometimes,” 4 to “Often,” and 5 to “Never.” The larger the value, the stronger the intensity of internet use among the rural older adults.

#### 3.2.3 Mediating variables

The mediating variable in this study is social participation, which refers to engaging in social activities during leisure time. Drawing on Peng and Ding’s (48) idea of selecting variables for social participation, the study chose the 12 items in the CGSS questionnaire that asked the respondents, “In the past year, did you often engage in the following activities during your free time?” The CGSS questionnaire asked the respondents “In the past year, how often did you engage in the following activities in your free time?,” “Watching TV or DVDs, going out to the movies, shopping, reading books/newspapers/magazines, participating in cultural activities such as listening to concerts and attending performances or exhibitions, meeting with relatives who do not live with you, gathering with friends, listening to music at home, taking part in sports, watching sports matches, doing handicrafts, and surfing the internet.” The original questionnaire answer options included “every day,” “several times a week,” “several times a month,” “several times a year,” and The original answers were reverse scored for ease of understanding, coded on a five-point Likert scale, i.e., “never” was assigned a value of 1; “several times a year or less” was operationalized as “never”; and “several times a year or less” was operationalized as “never.” “Occasionally” was assigned a value of 2; ‘Several times a month’ was assigned a value of 3; ‘Several times a week’ was assigned a value of 3; ‘Sometimes’ was assigned a value of 1; and ‘Occasionally’ was assigned a value of 1. “several times a week” is operationalized as ‘often participate’ and assigned the value of 4; ‘every day’ is operationalized as ‘participate every day’ and assigned the value of 5; and ‘every day’ is operationalized as ‘participate every day’ and assigned the value of 5 and operationalized “every day” as “participate every day” and assigned a value of 5.

In defining the mediator variables, the study first summed the means of the 12 items of social participation to form the total mediator variable indicator of social participation. Secondly, the 12 items were factor analyzed to obtain a KMO value of 0.720, and three main factors were extracted that explained 40.45% of the total variance. Rotation method: the Kaiser standardized maximum variance method, so the first 3 factors can be selected for analysis. According to the content of the activities, they were categorized into three types: leisure-entertainment type participation, cultural-activity type participation, and friend-gathering type participation. First, leisure-entertainment type participation includes watching TV, shopping, etc.; Second, cultural-activity type participation includes watching movies, reading books and newspapers, cultural activities, watching sports games, surfing the internet, etc.; Third, close friend-gathering type participation includes gathering with relatives who do not live together, gathering with friends, etc. The mean value of the question items in each type constitutes the three mediator variables under the index of overall social participation, and the larger the value represents that the older adults in the countryside. The larger the value, the more frequent the social participation of the rural older adults.

#### 3.2.4 Control variables

It has been shown that measures of mental health in older adults are also influenced by individual background characteristics, and the control variables selected for the study included gender (1 = male, 2 = female), age, level of education (for ease of computation, the answers to the original question items were recoded as 1 = elementary school and below, 2 = junior high school, 3 = high school or specialist, and 4 = university and above), and political status (for ease of computation, the answers to the original question items were recoded as 1 = member of the CPC, 0 = non-member of the CPC).

## 4 Results

### 4.1 Descriptive statistics

Definition and descriptive statistics of relevant variables after collation are shown in Table 1, the mean value of the explanatory variable rural older adults mental health is 3.679, indicating that the majority of rural older adults in the interviewed sample felt depressed or frustrated less frequently in the past 4 weeks, and that the rural older adults have a good state of mental health; and the mean value of the explanatory variable internet use is 1.877, indicating that a small number of rural older adults in the interviewed sample use the internet. At the level of mediating variables, the mean value of the overall social participation of the rural older adults is 1.923, indicating that most rural older adults participate in social activities occasionally; specifically, the mean value of the leisure-entertainment type participation is 3.248, the mean value of the cultural-activity type participation is 1.425, and the mean value of the friend-gathering type participation is 1.846, which indicates that the frequency of the rural older adults’s participation in social activities is low. At the level of control variables, the mean value of gender is 1.535, with slightly more women than men; the age span of the interviewed sample is 60–102 years old, with an average age of about 71 years old; the mean value of water education is 1.432, indicating that most of the rural older adults people’s education level is mostly elementary school and below; and the mean value of political status is 0.068, indicating that about 7% of the rural older adults people in the interviewed sample are members of the Communist Party.

**Table 1:** Variable definitions and descriptive statistics.

Variable type	Variable name	Variable definition and assignment	Minimum value	Maximum values	Average value	Standard deviation
Explained variables	Mental health	Degree of frustration and depression: always = 1, often = 2, sometimes = 3, rarely = 4, never = 5	1	5	3.679	1.181
Explanatory variables	Internet use	Frequency of use: never = 1, rarely = 2, sometimes = 3, often = 4, very often = 5	1	5	1.877	1.412
Mediating variables	Social participation	Degree of participation: Never = 1, Occasionally = 2, Sometimes = 3, Often = 4, Daily = 5	1	5	1.923	0.491
	Leisure-entertainment type participation	Degree of participation: Never = 1, Occasionally = 2, Sometimes = 3, Often = 4, Daily = 5	1	5	3.248	0.995
	Cultural-activity type participation	Degree of participation: Never = 1, Occasionally = 2, Sometimes = 3, Often = 4, Daily = 5	1	5	1.425	0.538
	Friend-Gathering type Participation	Degree of participation: Never = 1, Occasionally = 2, Sometimes = 3, Often = 4, Daily = 5	1	5	1.846	0.758
Control variables	Gender	Male = 1, Female = 2	1	2	1.535	0.499
	Age	Year (of crop harvests)	60	102	70.624	7.346
	Educational level	Elementary school and below = 1, middle school = 2, high school or college = 3, college and above = 4	1	3	1.432	0.653
	Political status	Non-Party members = 0, Communist Party members = 1	0	1	0.068	0.251

Perform variable description analysis on the 2028 selected samples.

### 4.2 Correlation analysis

At the level of variable correlation, according to the analysis of variable correlation coefficients in Table 2, firstly, there is a significant positive correlation between internet use and the mental health of rural older adults (*r* = 0.093, *p* < 0.001), which provides strong support for the validation of hypothesis H1. Secondly, there is a significant positive correlation between internet use and rural older adults social participation (*r* = 0.424, *p* < 0.001), which provides strong support for the validation of hypothesis H2; the social participation of rural older adults is significantly positively correlated with their mental health (*r* = 0.137, *p* < 0.001), which provides strong support for the validation of hypothesis H3. Similarly, there is a significant positive correlation between internet use and social participation of older adults people in rural areas; the social participation of rural older adults is significantly positively correlated with their mental health, which provides strong support for the validation of hypothesis H4. There is no significant correlation between internet use and leisure-entertainment type participation of rural older adults (*r* = −0.003, *p* > 0.05), but leisure-entertainment type participation is significantly positively correlated with the mental health of rural older adults (*r* = 0.119, *p* < 0.001), which provides strong support for the validation of hypothesis H4a. There is a significant positive correlation between internet use and cultural-activity type participation of rural older adults (*r* = 0.648, *p* < 0.001), and cultural-activity type participation and the mental health of rural older adults (*r* = 0.109, *p* < 0.001), which provides strong support for the validation of hypothesis H4b. There is a significant positive correlation between internet use and friend-gathering type participation of rural older adults (*r* = 0.102, *p* < 0.001), as well as between friend-gathering type participation and the mental health of rural older adults (*r* = 0.076, *p* < 0.001), which provides strong support for the validation of hypothesis H4c. In controlling variables, gender, age, education level, and political status are all significantly correlated with the mental health of rural older adults. Specifically, gender is significantly negatively correlated with the mental health of rural older adults (*r* = −0.128, *p* < 0.001); age is significantly negatively correlated with the mental health of rural older adults (*r* = −0.046, *p* < 0.05); there is a significant positive correlation between education level and the mental health of rural older adults (*r* = 0.132, *p* < 0.001); there is a significant positive correlation between political status and the mental health of rural older adults (*r* = 0.081, *p* < 0.001).

**Table 2:** Analysis of variable correlation coefficients.

	Mental health	Internet use	Social participation	Leisure-entertainment type participation	Cultural-activity type participation	Friend-gathering type participation	Gender	Age	Educational level	Political status
Mental health	1									
Internet use	0.093^***^	1								
Social participation	0.137^***^	0.424^***^	1							
Leisure-entertainment type participation	0.119^***^	−0.003	0.567^***^	1						
Cultural-activity type participation	0.109^***^	0.648^***^	0.740^***^	0.148^***^	1					
Friend-gathering type participation	0.076^***^	0.102^***^	0.473^***^	0.169^***^	0.197^***^	1				
Gender	−0.128^***^	−0.001	−0.094^***^	−0.075^***^	−0.103^***^	−0.023	1			
Age	−0.046^*^	−0.363^***^	−0.197^***^	−0.036	−0.257^***^	−0.029	−0.082^***^	1		
Educational level	0.132^***^	0.319^***^	0.275^***^	0.070^**^	−0.353^***^	0.047^*^	−0.236^***^	−0.308^***^	1	
Political status	0.081^***^	0.075^***^	0.137^***^	0.064^**^	0.144^***^	0.052^*^	−0.190^***^	0.039	0.180^***^	1

*p < 0.05, **p < 0.01, ***p < 0.001.

All of the above variables are significantly correlated with the explanatory variable mental health, so it is necessary to include them in subsequent regression analysis studies for in-depth exploration. At the same time, the above correlations only indicate that there is a two-by-two covariance between the variables, so further validation is needed in the regression analysis.

### 4.3 Regression analysis and results

At the main effect test level, the study explores the role of internet use in influencing the mental health of rural older adults. As can be seen from the results of regression analysis in Table 3, model 3 takes mental health as the outcome variable and four control variables, namely, gender, age, education level, and political status, as explanatory variables to explore its impact on the mental health of rural older adults; model 4, based on model 3, puts internet use as an independent variable into model 4 to analyze the role of internet use in influencing the mental health of the rural older adults, and the results indicate that internet use is significantly positively correlated with the mental health of rural older adults (*β* = 0.062, *p* < 0.05), hypothesis H1 is verified. Model 1 takes social participation as the outcome variable and four control variables, namely, gender, age, education level, and political status, as explanatory variables to explore their effects on social participation of rural older adults; Model 2, based on Model 1, puts internet use as an independent variable into Model 2 to analyze the role of the influence of internet use on the social participation of the rural older adults, and the results show that internet use is significantly positively correlated with the social participation of the rural older adults (*β* = 0.367, *p* < 0.001) are significantly positively correlated, hypothesis H2 is verified. Model 5 puts social participation as an independent variable into model 5 on the basis of model 3 to analyze the role of social participation in influencing the mental health of the rural older adults, and the results show that social participation is significantly positively correlated with the mental health of the rural older adults (*β* = 0.101, *p* < 0.001), the hypothesis H3 has been verified. Model 6 adds internet use as an independent variable to model 6 on the basis of model 5, and the results show that social participation has a significant effect on the mental health of rural older adults (*β* = 0.092, *p* < 0.001), and internet use is not significantly correlated with the mental health of rural older adults (*β* = 0.028, *P* > h0.05), and the effect of internet use of rural older adults is significantly correlated due to the presence of social participation. Changed, and it was preliminarily verified that social participation mediated the relationship between rural older adults internet use and their mental health, hypothesis H4 was preliminarily verified.

**Table 3:** Regression analysis of the impact of internet use on mental health and overall social participation among rural older adults.

Variant	Social participation			Mental health		
	Model 1	Model 2	Model 3	Model 4	Model 5	Model 6
Gender	−0.039	−0.053^*^	−0.099^***^	−0.102^***^	−0.095^***^	−0.097^***^
Age	−0.141^***^	−0.034	−0.028	−0.010	−0.014	−0.007
Educational level	0.205^***^	0.121^***^	0.091^***^	0.077^**^	0.070^**^	0.066^**^
Political status	0.098^***^	0.078^***^	0.047^*^	0.044	0.037	0.037
Internet use		0.367^***^		0.062^*^		0.028
Social participation					0.101^***^	0.092^***^
*R*^2^	0.101	0.211	0.030	0.033	0.039	0.040
Δ*R*^2^	0.099	0.209	0.028	0.030	0.037	0.037
*F*	56.919^***^	108.131^***^	15.518^***^	13.753^***^	16.397^***^	13.865^***^

*p < 0.05, **p < 0.01, ***p < 0.001. Regression coefficients in the table are standardized regression coefficients. Same as below.

According to the regression analysis in Table 4, there is no significant correlation between internet use and leisure-entertainment type participation of rural older adults in Model 2 (*β* = −0.037, *p* > 0.05). But in Model 5, there is a significant positive correlation between leisure-entertainment type participation of rural older adults and their mental health (*β* = 0.103, *p* < 0.001), Model 6 adds internet use as an independent variable to Model 5, and the results show that internet use has a significant impact on the mental health of rural older adults (*β* = 0.066, *p* < 0.01), Leisure-entertainment type participation has a significant positive correlation with the mental health of rural older adults (*β* = 0.105, *p* < 0.001), which preliminarily verifies the role of leisure-entertainment type participation in the relationship between internet use and mental health of rural older adults. Mediating effect, assuming that H4a has been preliminarily validated.

**Table 4:** Regression analysis of the impact of internet use on leisure-entertainment type participation and mental health among rural older adults people.

Variant	Leisure-entertainment type participation			Mental health		
	Model 1	Model 2	Model 3	Model 4	Model 5	Model 6
Gender	−0.059^*^	−0.058^*^	−0.099^***^	−0.102^***^	−0.093^***^	−0.096^***^
Age	−0.031	−0.042	−0.028	−0.010	−0.025	−0.005
Educational level	0.038	0.047	0.091^***^	0.077^**^	0.087^***^	0.072^**^
Political status	0.047^*^	0.049^*^	0.047^*^	0.044	0.042	0.039
Internet use		−0.037		0.062^*^		0.066^**^
Leisure-entertainment type participation					0.103^***^	0.105^***^
*R*^2^	0.011	0.012	0.030	0.033	0.040	0.044
Δ*R*^2^	0.009	0.010	0.028	0.030	0.038	0.041
*F*	5.777^***^	5.072^***^	15.518^***^	13.753^***^	16.933^***^	15.394^***^

According to the regression analysis in Table 5, there is a significant positive correlation between internet use and cultural-activity type participation of rural older adults in Model 2 (*β* = 0.598, *p* < 0.001), a significant positive correlation between cultural-activity type participation of rural older adults in Model 5 and their mental health (*β* = 0.063, *p* < 0.01), and a significant positive correlation between internet use and mental health of rural older adults in Model 6 based on Model 5. The results show that internet use and mental health of rural older adults (*β* = 0.037, *p* > 0.05), cultural-activity type participation and mental health of rural older adults (*β* = 0.041, *p* > 0.05) are not significant, indicating that hypothesis H4b has not been validated.

**Table 5:** Regression analysis of the impact of internet use on cultural-activity type participation and mental health among rural older adults people.

Variant	Cultural-activity type participation			Mental health		
	Model 1	Model 2	Model 3	Model 4	Model 5	Model 6
Gender	−0.036	−0.059^***^	−0.099^***^	−0.102^***^	−0.097^***^	−0.099^***^
Age	−0.179^***^	−0.005	−0.028	−0.010	−0.017	−0.010
Educational level	0.272^***^	0.135^***^	0.091^***^	0.077^**^	0.074^**^	0.071^**^
Political status	0.096^***^	0.064^***^	0.047^*^	0.044	0.041	0.041
Internet use		0.598^***^		0.062^*^		0.037
Cultural-activity type participation					0.063^**^	0.041
*R*^2^	0.160	0.452	0.030	0.033	0.033	0.034
Δ*R*^2^	0.158	0.451	0.028	0.030	0.031	0.031
*F*	96.141^***^	333.638^***^	15.518^***^	13.753^***^	13.824^***^	11.783^***^

According to the regression analysis in Table 6, it can be concluded that there is a significant positive correlation between internet use and friend-gathering type participation of rural older adults (*β* = 0.099, *p* < 0.001) in Model 2, and a significant positive correlation between friend-gathering type participation of rural older adults and their mental health (*β* = 0.067, *p* < 0.01) in Model 5. Model 6 adds internet use as an independent variable to Model 6 based on Model 5, and the results show that internet use is significantly correlated with the mental health of rural older adults (*β* = 0.056, *p* < 0.05), friend-gathering type participation is significantly correlated with the mental health of rural older adults (*β* = 0.062, *p* < 0.01). Hypothesis H4c has been preliminarily validated.

**Table 6:** Regression analysis of the impact of internet use on friend-gathering type participation and mental health among rural older adults people.

Variant	Friend-gathering type participation			Mental health		
	Model 1	Model 2	Model 3	Model 4	Model 5	Model 6
Gender	−0.009	−0.013	−0.099^***^	−0.102^***^	−0.099^***^	−0.101^***^
Age	−0.022	0.007	−0.028	−0.010	−0.026	−0.010
Educational level	0.030	0.007	0.091^***^	0.077^**^	0.089^***^	0.077^**^
Political status	0.046^*^	0.041	0.047^*^	0.044	0.044	0.041
Internet use		0.099^***^		0.062^*^		0.056^*^
Friend-gathering type Participation					0.067^**^	0.062^**^
*R*^2^	0.005	0.013	0.030	0.033	0.034	0.037
Δ*R*^2^	0.003	0.010	0.028	0.030	0.032	0.034
*F*	2.344	5.189^***^	15.518^***^	13.753^***^	14.333^***^	12.845^***^

### 4.4 Mediation effects test

**Figure 2:**
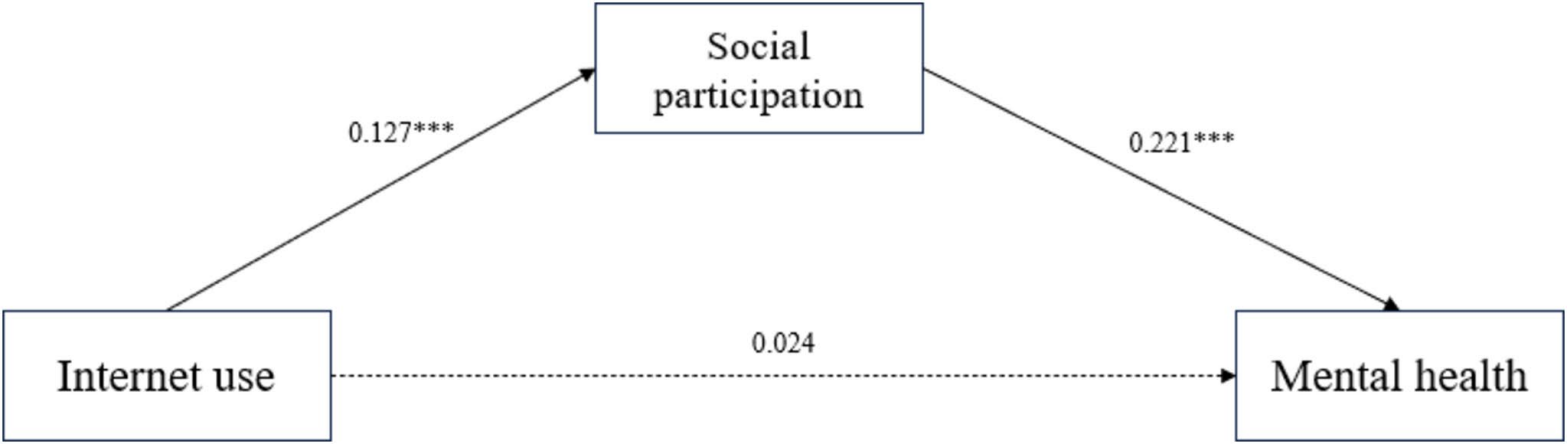
Path coefficients of internet use, social participation and mental health of rural older adults. ***p < 0.001.

**Figure 3:**
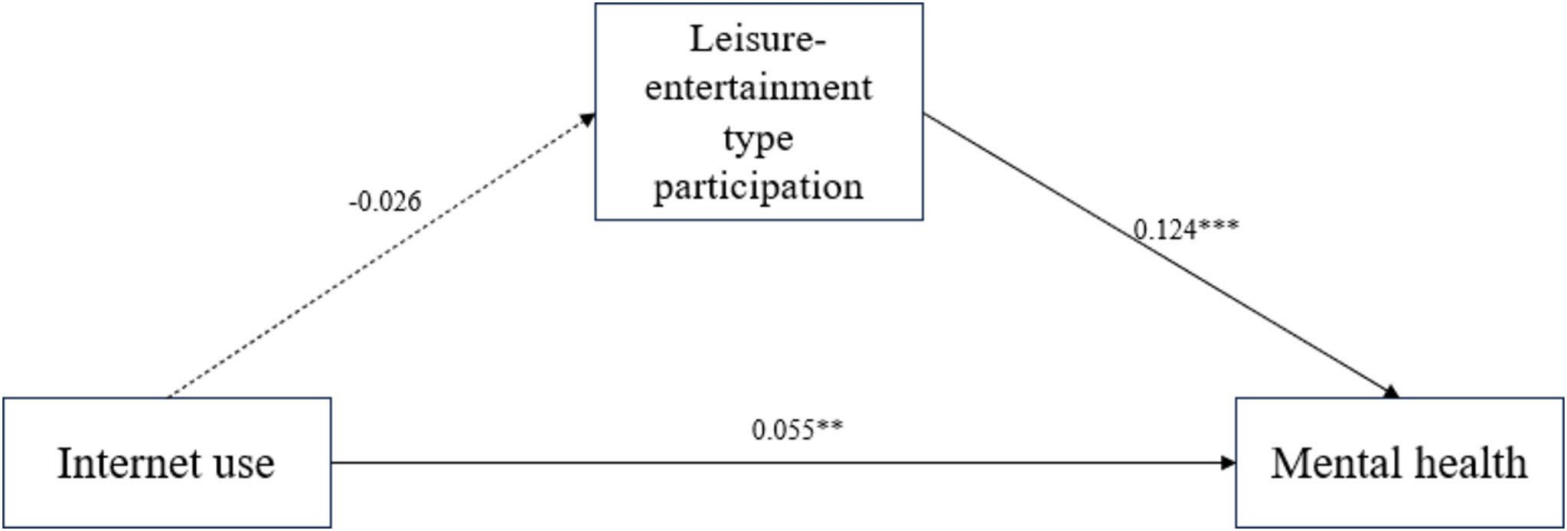
Path coefficient plots of internet use, leisure-entertainment type participation and mental health of rural older adults. **p < 0.01, ***p < 0.001.

**Figure 4:**
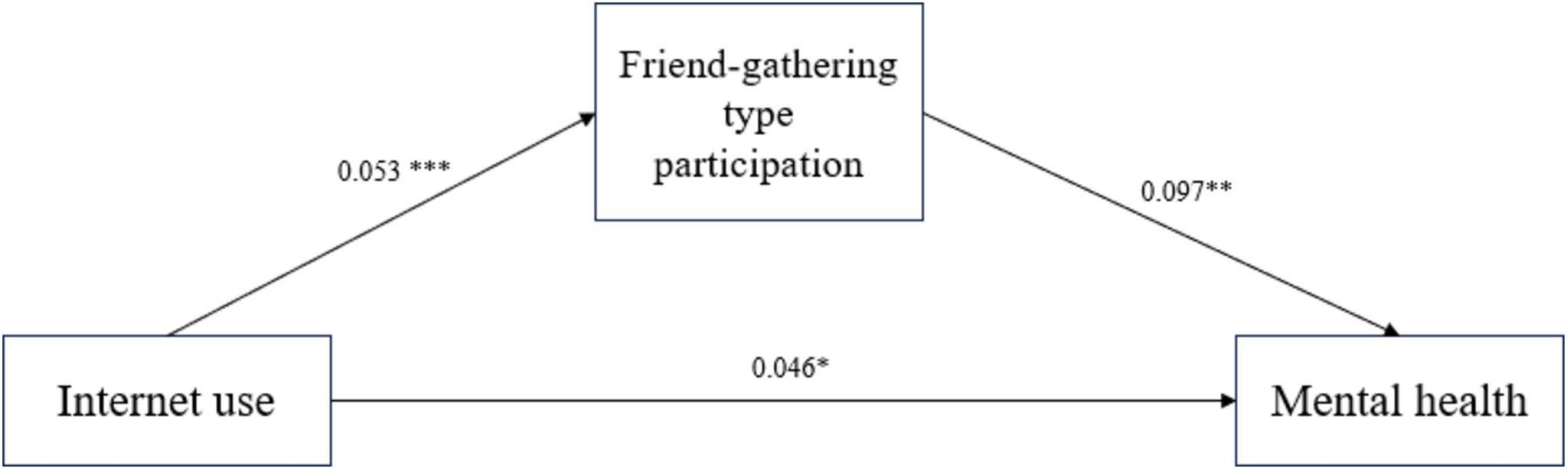
Path coefficient plots of internet use, friend-gathering type participation and mental health of rural older adults. *p < 0.05, **p < 0.01, ***p < 0.001.

In order to explore the internal mechanism of the significant positive effect of internet use on rural older adults’s mental health, social participation, leisure-entertainment type participation and friend-gathering type participation were further introduced as mediating variables to substitute into the model in the study. At the level of social participation mediation effect test, according to Hayes’ recommended method of testing mediation and moderating effects (49), and using his Process plug in developed based on SPSS, Model 4 was selected, and the Bootstrap method was used to validate and analyze the mediation effect of social participation, leisure-entertainment type participation and friend-gathering type participation on the relationship between internet use and rural older adults’s mental health. The Bootstrap sampling number was set at 5,000, and the confidence level of the confidence interval was set at 95%. Figure 2 showed the path coefficient between social participation and the variables of internet use and the mental health of rural older adults; Figure 3 showed the path coefficient between the variables of leisure-entertainment type participation in internet use and the mental health of rural older adults, from the path coefficient (Figure 3), it can be concluded that leisure-entertainment type participation cannot play a mediating role in the impact of internet use on the mental health of rural older adults. Therefore, hypothesis H4a has not been validated. Figure 4 showed the path coefficient between friend-gathering type participation and the variables of internet use and the mental health of rural older adults.

Next, social participation and friend-gathering type participation will be included in the mechanism of action for final mediation testing. According to the decomposition table of total, direct and mediating effects in Table 7, the total effect value of internet use on mental health of rural older adults was 0.052 (BC 95% confidence interval of 0.012–0.091). Although the direct effect value of internet use on mental health was 0.024 (Boots 95% CI [−0.019, −0.066]), this direct effect was not significant. However, the indirect effect through social participation had a value of 0.028 (Boots 95% CI [0.013, 0.045]) and this indirect effect was significant. Since the direct effect is not significant and the indirect effect is significant, the results suggest that overall social participation plays a fully mediating role between internet use and mental health of rural older adults, and therefore hypothesis H4 is tested.

**Table 7:** Breakdown of mediating effects of overall social participation.

	Efficiency value	SE	BC95% confidence interval		Effect size
			BootLLCI	BootULCI	
Total effect	0.052	0.020	0.012	0.091	
Direct effect					
Internet use → Mental health	0.024	0.022	−0.019	0.066	46.15%
Indirect effect					
Internet use → Social participation → Mental health	0.028	0.008	0.013	0.045	53.85%

As shown in Table 8, the decomposition of the mediating effect of friend-gathering type participation, the overall effect value of internet use on the mental health of rural older adults is 0.052 (Boots 95% CI [0.012, 0.091]). The direct effect of internet use on mental health was 0.047 (Boots 95% CI [0.007, 0.086]), which was significant; the indirect effect of friend-gathering participation was 0.005 (Boots 95% CI [0.001, 0.010]), and the indirect effect was also significant. The indirect effect was also significant. Therefore, the results suggest that friend-gathering type participation partially mediates the relationship between internet use and mental health among rural older adults, and hypothesis H4c was tested.

**Table 8:** Decomposition of the mediating effect of friend-gathering type participation.

	Efficiency value	SE	BC95% confidence interval		Effect size
			BootLLCI	BootULCI	
Total effect	0.052	0.020	0.012	0.091	
Direct effect					
Internet use → Mental health	0.047	0.020	0.007	0.086	90.38%
Indirect effect					
Internet use → Friend-gathering type participation → Mental health	0.005	0.002	0.001	0.010	9.62%

## 5 Discussion

This study explored the effect of internet use on the mental health of rural older adults, as well as the mediating role of social participation in the relationship between internet use and their mental health. Specifically, the primary finding of this study is a significant positive correlation between internet use and the mental health of rural older adults. This showed that the use of the internet can promote the mental health of rural older adults (30, 50), the more frequently and properly used the internet, the better their mental health (18–20). The popularization of the internet in rural areas can help older adults people in rural areas obtain information, engage in leisure activities, and communicate with others, broaden their horizons, enrich their personal cognition, and ultimately improve their quality of life (21, 22). For rural older adults people who are “digital refugees,” the internet is a life benefit they enjoy in their later years. Using the internet to post videos on media platforms to record their lives can help rural older adults improve their subjective well-being and mental health.

Additionally, we concluded that there is a significant positive correlation between internet use and social participation of rural older adults. This showed that the rural older adults actively use the internet, which helped to enhance their social participation. The internet is a positive representation of the re-socialization process of the older adults (51), as an online support platform for the older adults to participate in society, it is the premise for the older adults to engage in some offline social participation behavior, and can integrate the offline daily life of rural older adults with online behavior (36). Social participation is the embodiment of the life value of the older adults. The internet has constructed a new way of participation for the social activities of the older adults, fully affirmed the process of re socialization of the older adults and their social values, and helped the older adults to establish new social relations and enhance social participation (31).

Furthermore, we also found that there is a significant positive correlation between social participation and the mental health of rural older adults. This indicated that rural older adults who actively participated in social activities would have better mental health. Older people who actively participate in various social activities can fully utilize their socialization skills, and their psychological state will be healthier (52). The mental health status affects the lifestyle and attitude of rural older adults people. As a vulnerable group in society, rural older adults people are the objects of great care from society. Their mental health is not only their personal well-being, but also the greatest hope of their children for them (53). Rural older adults actively participating in social activities can improve their personal mental health level, which is also a vivid practice of “active aging” (11).

Finally, our study showed that social participation and friend-gathering participation under the category of social participation play a mediating role in the effect of internet use on the mental health of rural older adults. This mediating effect was manifested in the fact that social participation plays a fully mediating role in the effects of internet use on the mental health of rural older adults people; friend-gathering participation plays a partial mediating role in the effects of internet use on the mental health of rural older adults. This indicated that social participation was directly related to whether internet use can have an effect on the mental health of rural older adults (14). The social participation of rural older adults plays an important role in the entire social life, not only as an inner expression of individual participation, but also as a core component of older adults social capital, which has a positive integration effect on promoting interpersonal cooperation, economic development, and maintaining social order (54–56). Similarly, the importance of friend-gathering type participation in the use of the internet and the improvement of mental health status of the rural older adults has become increasingly prominent. Internet use can enhance the gathering frequency of the rural older adults and their relatives and friends, and the friend gathering of the rural older adults can enhance their social ties, thus promoting their mental health (44).

Based on the above research findings, this study believes that using the internet to improve the mental health level of rural older adults has important practical significance and requires joint assistance from multiple parties. First of all, the government departments should solve the problem that some rural areas have not yet covered the network hardware facilities according to the poverty alleviation measures, and at the same time expand the resettlement scope of the rural network signal coverage, so that the network can cover the whole village, provide the rural older adults with access to the internet, encourage the rural older adults to learn to use the internet, and improve the internet use rate.

Secondly, society should use the internet to strengthen the social participation behavior of rural older adults. This can be achieved by establishing senior universities for rural older adults people, offering digital skills training courses related to internet use, helping them become familiar with various internet skills, and organizing older adults people to participate in collective social activities through multiple channels of the internet, enriching their daily lives.

Finally, for rural older adults individuals, in the process of using the internet, they should maintain rational thinking, correctly identify the massive information in the network, protect personal privacy and information security, and strengthen their cognitive and practical abilities. Rural older adults people are forced to be involved in the digital wave, and at the same time, they need to accelerate digital feedback efforts to jointly protect the older adults internet user group, allowing rural older adults people to participate in the internet and obtain a higher quality of life.

In general, guiding and encouraging the rural older adults to use the internet, allowing them to accompany the internet and actively integrate into the digital society can not only improve their social participation level, but also significantly improve their mental health, which will help to achieve the grand goal of “active aging” and promote the harmonious development of society.

## 6 Conclusion

This study explored the effect of internet use on the mental health of rural older adults, as well as the mediating role of social participation and friend-gathering type participation. The two important results of the analysis are: (1) internet use is significantly and positively related to the mental health of rural older adults, and (2) internet use can have an indirect effect on the mental health of rural older adults through two pathways: (a) the complete mediating effect of social participation; (b) partial mediating role of friend-gathering type participation. In addition, this study utilized this latest development to propose some suggestions to strengthen the measures of internet use in promoting the mental health of rural older adults.

## 7 Limitations

It is important to acknowledge some limitations of this study. On the one hand, this study adopts a cross-sectional research design, which limits the causal explanatory power of the research results. Specifically, cross-sectional studies can only reveal the correlation between internet use and the mental health of rural older adults at a certain point in time, and cannot directly infer the causal relationship between the two. Future research can consider using longitudinal design or experimental research methods to track the internet use and mental health status of the same group of individuals at different time points, in order to more accurately evaluate the long-term effects of internet use on the mental health of rural older adults and provide stronger evidence for causal inference. On the other hand, from the perspective of variable selection, the measurement standard of internet use on the mental health of rural older adults is relatively simple, and the analysis of overall social participation and several participation behaviors under this specific type as intermediary variables lacks more universal significance. Future research should consider the measurement standards of internet use and mental health of rural older adults in a more in-depth and multi-dimensional manner, so as to enrich the measurement data of various variables in the follow-up research, so as to conduct more abundant research on the impact of internet use on the mental health of rural older adults.

## Data Availability

The datasets presented in this study can be found in online repositories. The names of the repository/repositories and accession number(s) can be found: https://pan.baidu.com/s/1kUJ0YBs3SLoxT35czZIz4w?pwd=6666.

## Footnotes

^1^ http://cgss.ruc.edu.cn/
